# Predicting complete cytoreduction for advanced ovarian cancer patients using nearest-neighbor models

**DOI:** 10.1186/s13048-020-00700-0

**Published:** 2020-09-29

**Authors:** Alexandros Laios, Alexandros Gryparis, Diederick DeJong, Richard Hutson, Georgios Theophilou, Chris Leach

**Affiliations:** 1 Department of Gynaecological Oncology, St James’s University Hospital, Leeds Teaching Hospitals, Leeds, LS9 7TF UK; 2 Unit of Endocrinology, Diabetes Mellitus and Metabolism, Aretaion Hospital, National and Kapodistrian University of Athens School of Medicine, Athens, Greece; 3 School of Human & Health Sciences, University of Huddersfield, Huddersfield, HD1 3DH UK; 4 Department of Psychology Services, South West Yorkshire Mental Health NHS Foundation Trust, The Laura Mitchell Health & Wellbeing Centre, Halifax, HX1 1YR UK

**Keywords:** Ovarian Cancer, Cytoreduction, Predictive factors, Machine learning, Artificial intelligence

## Abstract

**Background:**

The foundation of modern ovarian cancer care is cytoreductive surgery to remove all macroscopic disease (R0). Identification of R0 resection patients may help individualise treatment. Machine learning and AI have been shown to be effective systems for classification and prediction. For a disease as heterogenous as ovarian cancer, they could potentially outperform conventional predictive algorithms for routine clinical use. We investigated the performance of an AI system, the *k*-nearest neighbor (*k*-NN) classifier, to predict R0, comparing it with logistic regression. Patients diagnosed with advanced stage, high grade serous ovarian, tubal and primary peritoneal cancer, undergoing surgical cytoreduction from 2015 to 2019, was selected from the ovarian database. Performance variables included age, BMI, Charlson Comorbidity Index, timing of surgery, surgical complexity and disease score. The *k*-NN algorithm classified R0 vs non-R0 patients using 3–20 nearest neighbors. Prediction accuracy was estimated as percentage of observations in the training set correctly classified.

**Results:**

154 patients were identified, with mean age of 64.4 + 10.5 yrs., BMI of 27.2 + 5.8 and mean SCS of 3 + 1 (1–8). Complete and optimal cytoreduction was achieved in 62 and 88% patients. The mean predictive accuracy was 66%. R0 resection prediction of true negatives was as high as 90% using *k* = 20 neighbors.

**Conclusions:**

The *k*-NN algorithm is a promising and versatile tool for R0 resection prediction. It slightly outperforms logistic regression and is expected to improve accuracy with data expansion.

## Background

Ovarian, tubal and primary peritoneal cancer is the most lethal malignancy in women with 5-year survival not exceeding 30% [1]. The epithelial ovarian cancer (EOC) is the most frequent type representing 90% of all cases. Up to 60% of these cancers are diagnosed at an advanced stage (International Federation of Gynaecology and Obstetrics stage III and IV aEOC). Standard therapy comprises a combination of cytoreductive surgery and platinum-based chemotherapy, either as treatment following surgery (adjuvant) or as treatment both before and after surgery (neoadjuvant, NACT) [2]. Complete Cytoreduction (CCR) to no macroscopically visible residual disease (R0) is the mainstay of primary treatment. Cytoreductive outcome and tumor load are the most significant modifiable markers of survival [3, 4]. Following recent publications of landmark randomised studies demonstrating non-inferiority of NACT over primary surgery, it appears that NACT achieves higher R0 rates but, paradoxically, the survival rates are comparable [4]. To achieve macroscopic tumor clearance in peritoneally disseminated disease, maximal surgical effort is required, potentially including multi-visceral resection techniques, resulting in improved rates of cytoreduction [5].

Development of methods to predict surgical outcomes in addition to prognosis is an important paradigm in the realm of personalized medicine [6]. Optimal cytoreductive surgery for aEOC is pivotal for improving overall survival and disease-free survival. Developing methods to predict resectability of the disease will identify those who will benefit from maximal cytoreductive effort in a primary or interval surgical setting. Therefore, the risk of false negatives requires a final assessment of resectability as the first stage of cytoreductive surgery by laparotomy. Numerous composite models, including several aspects of preoperative work up and, sometimes, laparoscopy have been proposed to improve the accuracy of the predictive process [7].

Artificial intelligence (AI) has proven to have an enormous potential in many areas of healthcare with the added benefits of handling enormous amounts of biomedical data, coping with missing data and evolving in the presence of new data [8]. Machine learning (ML) is a branch of AI technology that allows computers to “learn” potential patterns from past examples. This approach has been used to predict cancer survival or optimal cancer drug therapies [9, 10].

Nevertheless, for a disease as heterogenous as ovarian cancer, accurate prediction is difficult with conventional statistics because patient characteristics show a multidimensional and non-linear relationship. There is evidence that ML methods can perform better in such setting than traditional statistical methods, familiar to clinicians, in handling complex information derived from large datasets with multiple input variables [11].

We aimed to investigate the performance of an ML system, the *k*-nearest neighbor (*k*-NN) classifier to predict R0 in high grade serous aEOC patients and compare it with conventional logistic regression. We hypothesised that certain predictors for R0 may work best for restricted subsets of these patients resulting in improved prediction accuracy. Therefore, the *k*-NN approach would identify those previously treated patients who most closely match the target patient (hence “nearest neighbors”) on intake variables. This strategy has been used to estimate the probabilities of alpine avalanches occurring [12]. It has been also used to predict treatment response to psychotherapy [13].

## Methods

We reviewed all patients diagnosed with histologically proven high grade serous only aEOC between Jan 2015 and Dec 2019 who were considered for cytoreductive surgery as part of their treatment pathway. All patients were managed at the Leeds Teaching Hospital Trust, Leeds, UK, which has been recently accredited by ESGO as a Centre of Excellence for ovarian cancer surgery. All patients were discussed at the central multi-disciplinary team (MDT) meeting and prospectively recorded in an electronic database. Patients were considered for cytoreductive surgery if the initial diagnostic workup (including physical examinations, hematological-biochemical examinations, tumor biomarkers, and CT scans) suggested a successful cytoreduction was feasible. Women either underwent primary debulking surgery (PDS) or 3–4 cycles NACT followed by interval debulking surgery (IDS) if: stage 4 disease; poor performance status; uncertainty about the possibility of optimal tumor removal. Women not exposed to surgery - those with progressive disease despite NACT, worsening performance status, and patient choice - were excluded. This retrospective observational cohort study was approved by the ethics review board.

K-NN models were applied to classify R0 vs non-R0 patients. In the *k*-NN approach, the intake variables are used only to identify the nearest neighbors for each specific subject, i.e. those subjects with intake variable values similar to the specific subject. Individual change is then predicted employing an unconditional growth model, using the average growth for the NNs as the prediction. Then, the optimum number of neighbors k was estimated based on the error calculation of the validation test. The higher *k*, the smoother the decision boundaries become. As *k* increases, we may end up in overfitting.

Specifically, we investigated models using three to 20 nearest neighbors. From the 147 patients who provided all observations in all required variables, we set 96 randomly chosen patients for the training set and the remaining 51 for the test set (i.e. a ratio of 65/35), and we repeated the process 500 times [14]. We compared the performance of *k*-NN models with that of multiple logistic regression using the same predictors in both approaches. For each iteration we estimated the prediction accuracy as the percentage of observations in the validation set that was correctly classified in each approach. Furthermore, for each iteration we estimated the percentage of true positives (i.e. patients with R0 correctly classified), true negatives (i.e. patients without R0 correctly classified), false positives and false negatives. In terms of predictors, for both approaches we used age, BMI, type of surgery, Charlson Comorbidity Index (CCI), Surgical Complexity Score (SCS), preTxCA125 and disease score. SCS was assigned based on the Aletti classification as low, intermediate and high. All quantitative variables were normalized before applying the *k*-NN models.

Categorical variables were presented as absolute and relative (%) frequencies. Continuous variables were presented using appropriate descriptive statistics (i.e. mean, median, SD, min, max). Quantitative variables were compared in R0 vs non-R0 patients using Mann-Whitney tests, while the association between R0 and non-R0 patients with qualitative variables was investigated using chi-square tests. All analyses were implemented in R statistical software using the library *class* and the *knn()* function (R Core Team (2017) [15] and IBM SPSS v. 25 (IBM Corp. Released 2017) [16].

## Results

A total of 154 high grade serous aEOC patients receiving treatment at Leeds Cancer Center were identified. Mean age and BMI were 64.4 + 10.5 yrs. and 27.2 + 5.8 respectively. The mean SCS was 3 + 1 (1–8). Of these patients, 31/154 (20%) underwent primary and 123/154 (80%) interval cytoreduction, respectively. Complete and optimal cytoreduction was achieved in 96/154 (62.3%) and 135/154 (87.7%) patients. The patients’ characteristics are summarised in Tables 1 and 2. From the variables selected to predict R0 resection, only disease score was significantly different between R0 and non-R0 patients (*p* = 0.0006).

**Table 1:** Descriptive statistics for the continuous variables, by group and overall

R0 vs non R0		N	Mean	Median	SD	Min	Max	***P***
**Age**	non R0	96	63.42	65.00	10.777	41	88	0.185
	R0	58	65.93	67.00	9.942	46	90	
	Total	154	64.36	66.00	10.509	41	90	
**BMI**	non R0	96	27.466	26.600	5.6198	18.3	51.4	0.474
	R0	58	26.884	25.800	6.0783	15.4	58.0	
	Total	154	27.247	26.400	5.7840	15.4	58.0	
**pre Tx CA125**	non R0	92	2202.95	732.50	4335.145	27	28,000	0.530
	R0	57	1560.68	586.00	2284.097	40	11,100	
	Total	149	1957.25	710.00	3691.556	27	28,000

**Table 2:** Absolute and relative frequencies for the categorical variables, by group and overall

	Levels	Non RO	RO	Totals	***P***
**RO category**		51 *(57.3%)*	38 *(42.7%)*	89	–
**Charlson Comorbidy Index (CCI)**	**4**	44 *(45.9%)*	32 *(55.2%)*	76 *(49.0%)*	**0.571**
	**5**	30 *(31.3%)*	12 *(20.7%)*	41 *(26.5%)*	
	**6**	14 *(14.5%)*	9 *(15.5%)*	23 *(14.8%)*	
	**7**	7 *(7.3%)*	3 *(5.2%)*	10 *(6.5%)*	
	**8**	1 *(1.0%)*	1 *(1.7%)*	2 *(1.3%)*	
	**9**	–	1 *(1.7%)*	1 *(0.6%)*	
	**Totals**	96	58	153	
**Surgical Complexity Score (SCS)**	**0**	54 *(56.3%)*	37 *(63.8%)*	91 *(58.7%)*	**0.333**
	**1**	30 *(31.3%)*	17 *(29.3%)*	47 *(30.3%)*	
	**2**	12	4	16 *(10.3%)*	
	**Totals**	96	58	154	
**Type of Surgery**	**Primary**	17 *(17.7%)*	14 *(24.1%)*	31 *(20.0%)*	**0.451**
	**Interval**	79 *(82.3%)*	44 *(75.9%)*	123 *(80.0%)*	
	**Totals**	96	58	154	
**Disease Score**	**1**	14 (14.6%)	2 *(3.4%)*	16 (10.3%)	**0.0006**
	**2**	60 *(62.5%)*	28 *(48.3%)*	88 *(56.8%)*	
	**3**	22 *(22.9%)*	28 *(48.3%)*	50 *(32.3%)*	
	**Totals**	96	58	154	

**Fig. 1:**
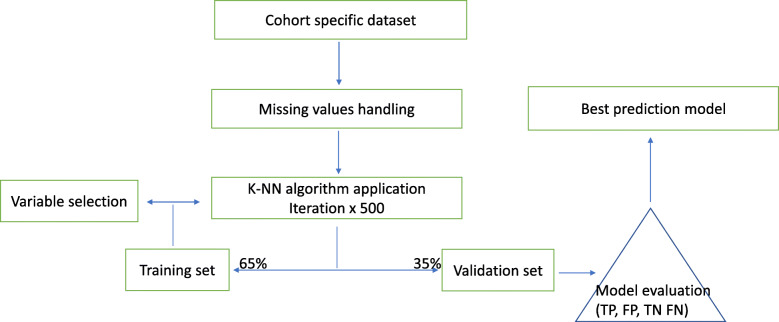
K-NN modelling framework flowchart: The framework for building the predictive model comprised three steps: data pre-processing, model training and performance evaluation. TP: true positive, FP: false positive, TN: true negative, FN: false negative

To predict R0 resection, the *k*-NN model was employed to classify patients in R0 versus non-Ro resection (Fig. 1). The classifier uses one tuning parameter (*k*) and is sensitive to data sampling and the number of neighbors *k*.

The predictive accuracy of the model for different choices of *k* is shown in Table 3. The highest mean predictive accuracy for *k*-NN methods was 65.8% (achieved for *k* = 15 and *k* = 19). The minimum predictive accuracy showed the lower bound of performance and hence the level of difficulty on the prediction problem in hand. The lowest performance bound was around 40% (*k* = 4) but the maximum predictive accuracy became as high as 82.4% for k = 12 and k = 15. Nevertheless, the *k*-NN model performed slightly better than logistic regression for the selected number of neighbors *k* (Table 3).

**Table 3:** Predictive accuracy of the k-NN model for different choices of the number of nearest neighbors × 500 replications and comparison with conventional logistic regression. Accuracy selects the best number of neighbors within a larger range and uses predictor importance when calculating distances

Number of nearest neighbors	Mean predictive accuracy (%)	Minimum predictive accuracy (%)	Maximum predictive accuracy (%)	Mean accuracy of TPs (%)	Mean accuracy of TNs (%)	Mean accuracy of FPs (%)	Mean accuracy of FNs (%)
3	57.5	41.2	72.5	37.0	70.7	29.3	63.0
4	57.8	39.2	78.4	**38.2**	70.6	29.4	**61.8**
5	60.7	43.1	74.5	36.4	76.3	23.7	63.6
6	60.8	41.2	82.4	37.5	75.8	24.2	62.5
7	62.9	45.1	78.4	35.2	80.6	19.4	64.8
8	62.8	43.1	78.4	35.7	80.2	19.8	64.3
9	64.5	47.1	80.4	34.5	83.6	16.4	65.5
10	64.4	47.1	78.4	35.0	83.2	16.8	65.0
11	65.2	45.1	78.4	34.6	84.7	15.3	65.4
12	64.7	43.1	**82.4**	34.8	83.9	16.1	65.2
13	65.5	45.1	80.4	34.1	85.6	14.4	65.9
14	65.3	45.1	80.4	33.7	85.5	14.5	66.3
15	**65.8**	47.1	**82.4**	32.5	87.1	12.9	67.5
16	65.4	47.1	80.4	32.0	86.8	13.2	68.0
17	65.5	**49.0**	80.4	30.5	88.0	12.0	69.5
18	65.5	47.1	76.5	29.8	88.3	11.7	70.2
19	**65.8**	47.1	80.4	28.8	89.4	10.6	71.2
20	65.6	43.1	80.4	28.1	**89.5**	**10.5**	71.9
**Logistic regression results**	**Mean predictive accuracy (%)**	**Minimum predictive accuracy (%)**	**Maximum predictive accuracy (%)**	**Mean accuracy of TPs (%)**	**Mean accuracy of TNs (%)**	**Mean accuracy of FPs (%)**	**Mean accuracy of FNs (%)**
–	63.4	2.0	80.4	42.7	76.7	23.1	57.1

**Fig. 2:**
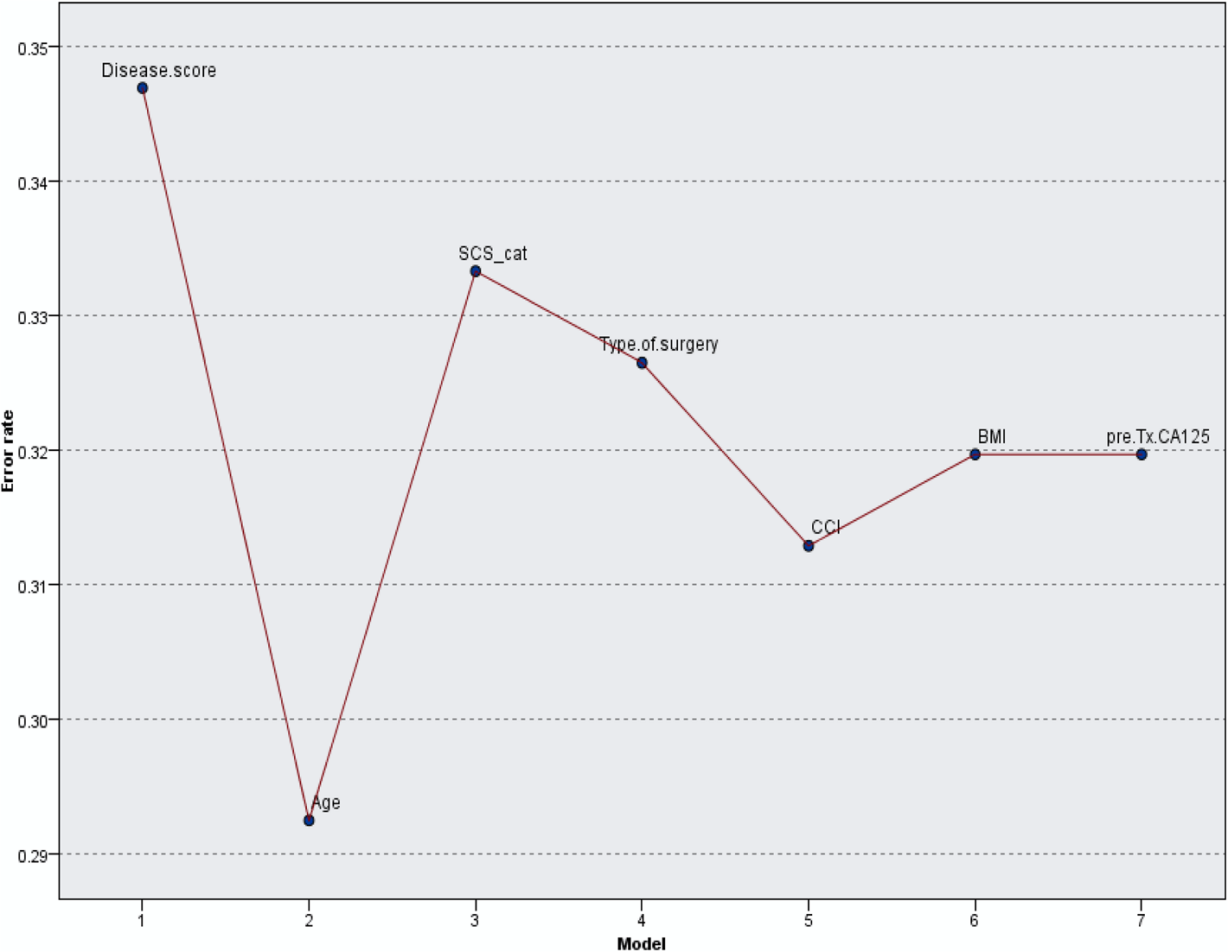
Variable importance chart. Misclassification error for each predictor from the *k*NN model for *k* = 15. Selected predictors are weighted by their relative importance for R0 resection prediction in the training set

In addition to performance comparison, we analysed the most important variables that contributed to the prediction model (R0 resection). The relative importance of selected clinical variables was quantified by calculating the prediction accuracy/error rate in relation to the number of predictors included in the models. Figure 2 displays the misclassification error of selected predictors in the training set for a given neighborhood size. For *k* = 15, R0 resection is best predicted by a *k*NN model that includes age and disease score only. Notably, if surgical complexity score is added, the model performs worse with R0 resection prediction accuracy just above 78%.

Table 4 demonstrates a prediction comparison for CCR between the *k*NN model and logisitic regression on a random sample of 20 patients from the training set, who underwent R0 resection. It also provides information on the potential clinical use of the predictive NN strategy to counsel individual patients about their surgery. Patient 2 was a 66-year-old woman, slightly overweight, who returned to outpatients for discussion about interval debulking surgery. She had a history of ischaemic heart disease. Her pretreatment CA125 was 466 u/mL. She had a good partial response to chemotherapy and on imaging, her disease appeared limited to the pelvis. In her case, complete cytoreduction could be predicted from two to four previously treated patients with similar BM, limited or no co-morbidities, who underwent interval cytoreduction. Selecting information from more previously treated patients of the same age group would aid R0 resection prediction even if a standard cytoreductive surgery was offered. (Table 4).

**Table 4:** R0 resection prediction comparison between the *k*NN model and logistic regression on a random sub cohort of 20 patients from the training set. All these patients underwent primary or interval debulking surgery and complete cytoreduction was achieved

Patient	Age	BMI	Charlson Comorbidity Index	Type of surgery	Surgical Compexity Score categories	Pre Tx Ca125	Disease score	kNN model	Logistic regression
**1**	52	26.4	5	Interval	Standard	10,156	1	R0	R0
**2**	66	28.1	5	Interval	Standard	466	1	R0	R0
**3**	77	18.8	4	Interval	Standard	1876	2	R0	R0
**4**	58	28.7	4	Interval	Radical	1900	2	R0	R0
**5**	64	32.6	5	Interval	Ultraradical	1211	3	R0	R0
**6**	51	23.8	4	Interval	Radical	1121	2	R0	R0
**7**	52	39	4	Interval	Standard	586	2	R0	R0
**8**	68	24.8	5	Interval	Standard	488	2	R0	R0
**9**	48	31.9	5	Interval	Standard	3300	2	R0	R0
**10**	88	23	5	Interval	Standard	286	1	Non-R0	R0
**11**	57	26.9	4	Primary	Standard	875	2	R0	R0
**12**	57	38.8	5	Interval	Radical	1765	2	R0	R0
**13**	69	28	4	Interval	Standard	1417	1	R0	R0
**14**	68	28.7	6	Interval	Standard	52	2	Non-R0	Non-R0
**15**	50	33.9	4	Interval	Standard	2454	2	R0	R0
**16**	55	33.2	4	Interval	Standard	1612	2	R0	R0
**17**	68	21.6	4	Interval	Ultraradical	1800	2	R0	R0
**18**	75	33.3	6	Interval	Standard	87	2	Non-R0	Non-R0
**19**	73	24.7	4	Primary	Radical	181	2	R0	R0
**20**	57	19.5	4	Primary	Ultraradical	1176	2	R0	R0

## Discussion

In EOC, it is widely accepted that R0 resection following cytoreductive surgery is associated with the best overall outcomes [17]. For a disease as heterogenous as EOC, a standard “one-size fits all” approach for surgical cytoreduction cannot be acceptable. Predicting surgical outcomes and stratifying patients with respect to CCR based on clinical information is fundamental towards individualised optimal cancer care. This study describes the development of an ML model, which can be directly relevant to aEOC patients and their treating surgeons. The model uses available data items as input variables. These features can be readily available to the surgeons before performance of a laparotomy. Equally, it can be used at the research setting when reliable outcome data soon after surgery are required without long follow-up periods.

To predict CCR, our aim was to make predictions based on similar surgical outcomes from already-treated patients. The *k*-NN approach mirrors the way clinicians often talk about how they use their clinical experience to treat their patients.

Accurate prediction of CCR could allow identification of those patients, who, following primary chemotherapy, would not benefit from surgery and they should be considered for chemotherapy continuation. In that respect, the model would be useful for ongoing decision making and quality assurance during their cancer treatment pathway. Incomplete cytoreduction can be predicted with very high positive predictive value, close to 100% (i.e, if the test predicts residual disease regardless of the size, the R0 resection status will not be achieved) [18]. Nonetheless, a method to predict CCR with high likelihood (i.e. if the test predicts that no residual disease is achievable it will be achieved- the true negative) is still required. To serve this purpose, the major finding of this study is the prediction of CCR with a mean accuracy of true negatives as high as 90% for a tuning number of neighbors *k* = 20 (Table 3).

The *k*-NN method is grounded in the idea that, to predict R0 resection following cytoreductive surgery, accuracy is more valid if one uses homogeneous subsamples. The more we know about a patient can potentially affect our surgical effort but, the more patients available, the more valuable the search for similar patients and the better the prediction of R0 resection. Such information can be particularly important to the elderly patients; as their cancer progresses or they experience increased comorbidities with age, often are too frail to be considered for cytoreductive surgery. Not infrequently, they question the effect of surgery on them or their subjective perception of pre-operative wellbeing is poor. A previous “good clinical experience” on surgical outcomes can be reassuring and may help refine risk stratification. Equally, pre-operative prediction of required surgery can focus on disease resectability rather than surgical complexity.

The main strength of the *k*NN approach is versatility. For instance, if the predictive method is planned to be used in a cancer screening system, specificity (false negatives) should be high; in a diagnostic system, both sensitivity (true positives) and specificity (true negatives) should be high. Testing the performance based on the number of nearest neighbors without choosing a classifier threshold allows us to keep all these applications feasible. In this way, many potential clinical applications should be captured by this model.

The mean predictive model accuracy was 66%. Above 65% this is still satisfactory, but a number closer to 75% would have been preferable. One reason may be the fact that datasets based on clinical practice are often incomplete. If one variable value is missing, the entire case cannot be used in building the ML model. In our study, there was a very small however, amount of missing data. This limitation can be overcome by median imputation of the missing data. Another reason may be the high correlation amongst the variables that may render the model partly unstable due to collinearity (which further exists when the variables are increased). Use of fewer prediction variables would have rendered better accuracy at the cost of not collectively addressing the complexity of cytoreduction, leading to a less meaningful interpretation of outcome data. In the realm of precision medicine, the ability of ML models to discover embedded patterns within data by handling multiple factors at once, irrespective of the data size may lead to a better understanding of the complexity in achieving CCR. Cohort expansion to a larger sample size is expected to improve predictability. Prospective internal validation of the model to a larger cohort is currently ongoing.

Our results also suggest that the *k*-NN predictions of CCR slightly outperform those predicted by conventional statistics. That is, the strategy where predictions are based on small subsamples of patients with similar clinical characteristics appears superior to the strategy of basing predictions on optimally weighted combinations of clinical characteristics. This is likely due to extraction of homogeneous samples with high similarity to any individual patient. The study sample was not large but offered enough similar neighbors on average to allow for accurate predictions. Use of *k*-NN as a tuning parameter gives non inferior results compared to multiple logistic regressions, indicating that R0 prediction in aEOC can be made stable irrespective of prediction models.

From the variables tested to contribute in R0 prediction, only disease score was statistically significant. Nevertheless, this would not affect *k-*NN model accuracy; it is the relative importance of each predictor in estimating the model. Selected predictors were weighted equally against each other in selecting NNs and may have played a role in the success in predicting the surgical outcome. Some variables may be differentially weighted in the NN analysis to reflect estimates of their contribution to relevant similarity (Fig. 2). Variable importance does not necessarily relate to model accuracy. It relates to the importance of each variable in making a prediction, not whether the prediction is accurate.

Other studies used an ordinal classification method to predict surgical outcomes in aEOC patients with a 64.9% accuracy and AUC of 0.697 (R0 vs non-R0) based solely on preoperative information [19]. Another AI model predicted the outcome of surgery and again showed that ANN could predict outcome (optimal cytoreduction vs. suboptimal cytoreduction) with 77% accuracy and an AUC of 0.73. Application of AI weighted the importance of factors predicting CCR at secondary cytoreductive surgery for recurrent ovarian cancer [20].

This retrospective study was a single institution experience with limited heterogeneity in the study population, which may differ from other tertiary unit settings. It was not powered to test how many overall cases are necessary to define sufficiently homogeneous subgroups of NNs, which are adequate for predictions. Nevertheless, ML retains the strength of the structural model used for the R0 prediction even when applied in other populations and reveal different prediction features.

## Conclusions

We considered the problem of predicting CCR in aEOC patients using clinical preoperative and intraoperative variables and focused our analysis on the comparison between AI and conventional regression models under the same resampling conditions. The study demonstrated the feasibility of using the *k*-NN approach, which is very much reflective of “previous clinical experience” for accurate prediction of R0 resection during aEOC surgery. The model slightly outperforms conventional logistic regression. It should be further improved with data expansion and become directly available to clinicians.

## Data Availability

The data that support the findings of this study are available from the corresponding author upon reasonable request.
